# Mechanisms, Significance, Detection and Emerging Therapies for Carbapenem‐Resistant Enterobacterales: A Scoping Review

**DOI:** 10.1155/jotm/5663176

**Published:** 2026-10-05

**Authors:** Oluchi Judith Osuala, Chioma Miracle Ojiako, Chioma C. Ezeuko, Kasso Terhemen, Constance Chinyere Ezemba, Victor O. Etikudike, Charles O. Nnadi, Angus Nnamdi Oli

**Affiliations:** ^1^ Department of Pharmaceutical Microbiology and Biotechnology Madonna University Elele Rivers Nigeria; ^2^ School of Science, Technology and Engineering University of the Sunshine Coast 90 Sippy Downs Drive Sippy Downs Queensland 4556 Australia usc.edu.au; ^3^ Microbiology Program, College of Science, Technology, Engineering and Mathematics Alabama State University Montgomery Alabama 36104 USA alasu.edu; ^4^ Department of Obstetrics and Gynaecology University of Port-Harcourt Port-Harcourt Nigeria unicamp.br; ^5^ Department of Nursing Science Marsha Fuerst School of Nursing Riverside California USA; ^6^ Department of Microbiology Chukwuemeka Odumegwu Ojukwu University Uli Anambra Nigeria coou.edu.ng; ^7^ Department of Pharmaceutical and Medicinal Chemistry University of Nigeria Nsukka Enugu Nigeria unn.edu.ng; ^8^ Department of Pharmaceutical Microbiology and Biotechnology Nnamdi Azikiwe University Awka Anambra Nigeria unizik.edu.ng

**Keywords:** β-lactamases, antimicrobial resistance, antimicrobial therapy, carbapenem-resistant Enterobacterales (CRE), carbapenemase-producing Enterobacterales (CPE), carbapenemases, molecular diagnostics, multi–drug-resistant Gram-negative bacteria

## Abstract

**Background:**

Carbapenems are among the most important broad‐spectrum β‐lactam antibiotics for the treatment of severe infections caused by multi–drug‐resistant Gram‐negative bacteria. However, the global emergence and dissemination of carbapenem‐resistant Enterobacterales (CRE), particularly carbapenemase‐producing Enterobacterales (CPE), have significantly compromised their clinical utility. Resistance is primarily driven by the acquisition of genes encoding carbapenemases, often in combination with porin alterations, efflux pump overexpression and AmpC hyperproduction, resulting in limited treatment options and increased morbidity and mortality.

**Objectives:**

This scoping review aimed to synthesise current evidence on the molecular mechanisms of carbapenem resistance, the classification and epidemiology of carbapenemases, advances in diagnostic approaches and emerging therapeutic strategies for CRE infections.

**Methods:**

A scoping review was conducted in accordance with the PRISMA extension for Scoping Reviews (PRISMA‐ScR). Literature published between January 2005 and March 2026 was identified through systematic searches of PubMed/MEDLINE, Scopus and Google Scholar. Eligible studies included original research articles, surveillance reports, systematic reviews and clinical guidelines addressing carbapenem resistance mechanisms, carbapenemase epidemiology, diagnostics or treatment strategies. Data were extracted and synthesised using a narrative and thematic approach.

**Results:**

Carbapenemase production was identified as the predominant mechanism of resistance among CRE, with KPC, NDM, OXA‐48‐like, VIM and IMP enzymes driving global dissemination. Considerable geographical variation exists in carbapenemase distribution, reflecting differences in antimicrobial use, surveillance capacity and healthcare infrastructure. Advances in molecular diagnostics, including polymerase chain reaction, loop‐mediated isothermal amplification, whole‐genome sequencing and rapid immunochromatographic assays, have improved detection and surveillance. Emerging therapeutic options, particularly novel β‐lactam/β‐lactamase inhibitor combinations and cefiderocol, have expanded treatment possibilities, although resistance to newer agents continues to emerge.

**Conclusions:**

CREs remain a major global public health threat. Strengthened surveillance systems, improved access to rapid diagnostics, carbapenemase‐directed therapeutic strategies and continued antimicrobial development are essential to limit the spread of resistance and improve clinical outcomes.

## 1. Introduction

Carbapenems remain among the most potent β‐lactam antibiotics for the treatment of life‐threatening infections caused by multi–drug‐resistant (MDR) Gram‐negative bacteria. Their distinctive molecular architecture, including a trans‐configured β‐lactam fused to a five‐membered ring and resistance to hydrolysis by most β‐lactamases, confers exceptional stability and broad‐spectrum activity against extended‐spectrum β‐lactamase (ESBL) and AmpC‐producing organisms [1]. Carbapenems exert bactericidal activity through high‐affinity binding to essential penicillin‐binding proteins (PBPs), predominantly PBP 1 and PBP 2 [2].

Of all the carbapenem‐resistant Gram‐negative bacteria, carbapenem‐resistant Enterobacterales (CRE) and especially carbapenemase‐producing Enterobacterales (CPE) are some of the most pressing public health challenges owing to their rapid spread through horizontal gene transfer, wide drug resistance, multiple resistance mechanisms and high associated mortality rates [3–5]. Although carbapenem resistance is also highly prevalent among nonfermenting Gram‐negative pathogens such as *Pseudomonas aeruginosa* and *Acinetobacter baumannii*, CRE remain the predominant drivers of the global carbapenem resistance crisis owing to their widespread dissemination across healthcare settings and communities and their exceptional ability to acquire and disseminate mobile resistance determinants [6, 7].

Carbapenemases are classified under the Ambler scheme into Class A serine carbapenemases, including *Klebsiella pneumoniae* carbapenemase (KPC); Class B metallo‐β‐lactamases (MBLs), including New Delhi metallo‐β‐lactamase (NDM), Verona integron–encoded metallo‐β‐lactamase (VIM) and imipenemase (IMP); and Class D oxacillinases (OXA‐type enzymes) [8]. Among the Enterobacterales, *Klebsiella pneumoniae* occupies a uniquely important position in the epidemiology of carbapenem resistance. The species is strongly associated with high‐risk epidemic clones, including sequence types ST258, ST11, ST147 and ST307, which have played central roles in the global dissemination of KPC, NDM and OXA‐48‐like carbapenemases [3]. Its highly adaptable genome, capacity to acquire mobile genetic elements, ability to colonise the gastrointestinal tract asymptomatically, persistence in healthcare environments and propensity for biofilm formation have established *K. pneumoniae* as the principal reservoir and disseminator of carbapenemase genes among clinically important Enterobacterales. Consequently, this pathogen has become a major driver of the contemporary CRE epidemic and remains a primary target for surveillance and infection prevention efforts [3, 5].

The clinical management of CRE infections has become increasingly complex due to the emergence of resistance to traditional last‐line agents, including colistin and tigecycline, as well as the continued evolution of novel carbapenemase variants. Although substantial advances have been achieved through the development of rapid molecular diagnostic platforms and newer β‐lactam/β‐lactamase inhibitor combinations, significant challenges remain in the timely detection, surveillance, treatment and containment of carbapenemase‐producing organisms (CPOs). Furthermore, marked geographical variation exists in the prevalence and molecular epidemiology of carbapenemases, with surveillance systems and diagnostic capabilities remaining highly unequal between high‐income and low‐ and middle‐income countries, leading to considerable underreporting in many regions [9, 10].

Given the continuing global expansion of KPC‐, NDM‐, OXA‐48‐, VIM‐ and IMP‐producing Enterobacterales, there is an urgent need to consolidate current knowledge regarding the molecular mechanisms underlying carbapenem resistance, the epidemiology of carbapenemase dissemination, advances in diagnostic technologies and emerging therapeutic options. This review synthesises current knowledge on carbapenem pharmacodynamics, the molecular basis of resistance, epidemiologic patterns of CPOs, advances in molecular diagnostics and evolving therapeutic strategies. The objective is to delineate the scale of the global carbapenem resistance threat and underscore the urgent need for coordinated multifaceted containment efforts.

## 2. Methodology

### 2.1. Review Objective and Scope

This review aimed to map and critically synthesise current evidence regarding molecular mechanisms, epidemiology, diagnostic approaches and emerging therapeutic strategies associated with CREs and other clinically significant carbapenem‐resistant Gram‐negative bacteria. The review further evaluated the global dissemination of carbapenemase genes such as KPC, NDM, OXA‐48, VIM and IMP, as well as current advances in antimicrobial management.

### 2.2. Study Design

This study was conducted as a scoping review following the Preferred Reporting Items for Systematic Reviews and Meta‐Analyses extension for Scoping Reviews (PRISMA‐ScR) framework. To ensure methodological rigour and transparency, the review process was guided by the PRISMA statement principles where applicable [11]. The scope encompassed carbapenem pharmacology, molecular mechanisms of resistance, epidemiology of carbapenem‐resistant Gram‐negative pathogens, diagnostic modalities and current therapeutic strategies. Eligible literature published between January 2005 and March 2026 was considered for inclusion in order to capture both foundational and contemporary developments in carbapenem resistance research. The search period was selected to encompass the emergence and global dissemination of major carbapenemase families, including KPC, NDM, OXA‐48‐like, VIM and IMP enzymes, while capturing contemporary developments in diagnostics and antimicrobial therapy.

### 2.3. Search Strategy

A comprehensive and systematic literature search was conducted across multiple electronic databases, including PubMed/MEDLINE, Scopus and Google Scholar. Relevant publications were identified using combinations of Medical Subject Headings (MeSH) and free‐text keywords related to carbapenem resistance, CPE, molecular resistance mechanisms, diagnostic approaches and therapeutic strategies. Search terms included combinations of ‘carbapenem resistance’, ‘carbapenemase‐producing Enterobacterales’, ‘carbapenem‐resistant Gram‐negative bacteria’, ‘KPC’, ‘NDM’, ‘OXA‐48’, ‘metallo‐β‐lactamases’, ‘β‐lactamase inhibitors’, ‘porin loss’, ‘efflux pumps’, ‘molecular diagnostics’, ‘ceftazidime‐avibactam’, ‘cefiderocol’, ‘aztreonam‐avibactam’ and ‘combination therapy’. Boolean operators (AND, OR) were used to combine search concepts and refine retrieval of relevant studies.

To maximise the completeness of the review and minimise the risk of omitting relevant evidence, the electronic database search was supplemented by manual screening of reference lists from eligible primary studies, review articles, meta‐analyses, international surveillance reports and clinical practice guidelines. Searches were restricted to articles published in English between January 2005 and March 2026 to capture both foundational studies and contemporary developments in the epidemiology, diagnosis and management of carbapenem‐resistant organisms.

### 2.4. Eligibility Criteria

#### 2.4.1. Inclusion Criteria

Studies were considered eligible for inclusion if they addressed one or more aspects of carbapenem antibiotics, carbapenem resistance mechanisms, carbapenemase epidemiology, diagnostic approaches or therapeutic strategies for carbapenem‐resistant Gram‐negative pathogens. Particular emphasis was placed on studies investigating CRE, including *Klebsiella pneumoniae*, *Escherichia coli*, *Enterobacter* spp. and other clinically relevant Enterobacterales. Where relevant to the understanding of carbapenem resistance epidemiology, molecular mechanisms or therapeutic developments, studies involving other important carbapenem‐resistant Gram‐negative pathogens, such as *Pseudomonas aeruginosa* and *Acinetobacter baumannii*, were also considered.

Eligible publications included original peer‐reviewed research articles, systematic reviews, meta‐analyses, international surveillance reports and authoritative clinical practice guidelines. Studies were required to provide data relating to molecular epidemiology, carbapenemase genes, resistance mechanisms, diagnostic methodologies, antimicrobial susceptibility, therapeutic interventions or clinical outcomes associated with carbapenem‐resistant organisms. Only articles published in English were included.

#### 2.4.2. Exclusion Criteria

Studies were excluded if they were duplicate records identified across multiple databases, contained insufficient methodological detail or reported findings unrelated to carbapenem resistance. Conference abstracts, editorials, commentaries, book reviews and grey literature without accessible full‐text articles were excluded because of limited methodological transparency and data availability. Publications focussing exclusively on antimicrobial resistance mechanisms unrelated to carbapenem resistance were also excluded. Additionally, studies lacking sufficient microbiological, epidemiological, diagnostic or therapeutic relevance to the objectives of this review were not considered for inclusion.

### 2.5. Study Selection Process

Literature records retrieved from the database searches were compiled and exported into a reference management system where duplicate records were identified and removed electronically and manually. Two reviewers independently screened the remaining titles and abstracts against the predefined eligibility criteria. Subsequently, full‐text articles of potentially relevant records were retrieved and rigorously evaluated for final inclusion. Discrepancies during the screening and selection phases were resolved through consensus or consultation with a third reviewer. The literature search identified 216 records across the selected databases and supplementary reference screening. Following removal of 34 duplicate records, 182 records underwent title and abstract screening, of which 96 were excluded. Eighty‐six full‐text articles were assessed for eligibility, and 24 studies were excluded based on predefined criteria. Ultimately, 62 studies met the inclusion criteria and were incorporated into the final synthesis. The study selection process is summarised in the PRISMA flow diagram (Figure 1).

**FIGURE 1:**
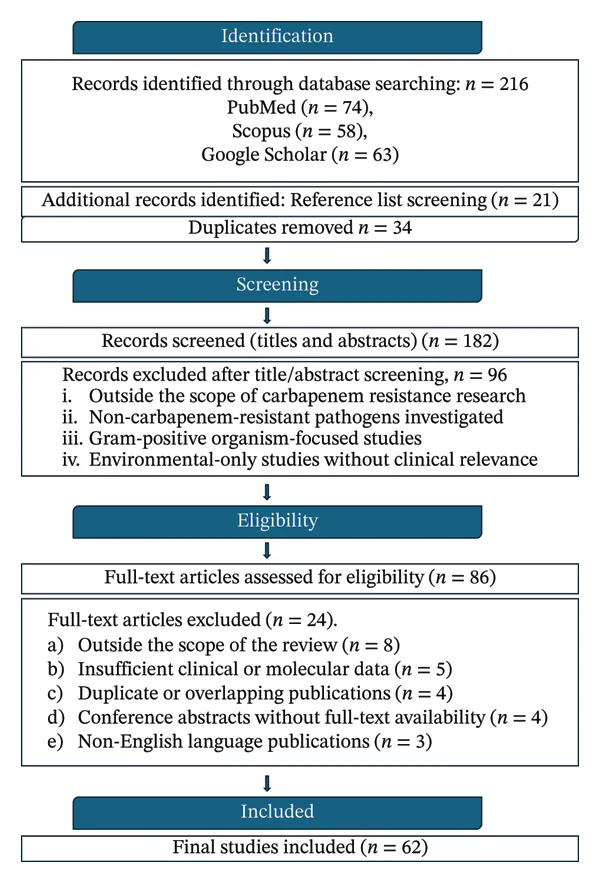
PRISMA‐ScR flow diagram of the literature selection process illustrating the stages of database identification, title and abstract screening, full‐text eligibility assessment and final study inclusion.

### 2.6. Data Extraction and Management

Data from all eligible studies were systematically extracted using a standardised data extraction template developed specifically for this review. Information collected from each study included bibliographic details (first author, year of publication and journal source), study characteristics (geographical location, clinical setting, study design and target bacterial species) and microbiological findings, including carbapenem resistance mechanisms, carbapenemase genes identified, porin alterations, efflux pump expression and antimicrobial susceptibility profiles. Additional data relating to diagnostic methodologies, including phenotypic and molecular detection approaches, as well as therapeutic interventions and clinical management strategies, were also extracted. Outcome measures recorded included principal study findings, epidemiological trends, clinical outcomes, treatment responses, surveillance observations and authors’ conclusions. Data extraction was performed to ensure consistent capture of information relevant to the objectives of the review and to facilitate comparison across studies.

### 2.7. Data Synthesis

Given the diversity of study designs, geographical settings, diagnostic methodologies and outcome measures identified during the literature search, a qualitative narrative and thematic synthesis approach was adopted. Extracted evidence was organised into six major thematic domains: (i) pharmacology and mechanisms of action of carbapenems; (ii) molecular and biochemical mechanisms of carbapenem resistance; (iii) classification, diversity and global epidemiology of carbapenemases; (iv) dissemination and resistance patterns among CPEs and other clinically relevant Gram‐negative pathogens; (v) phenotypic, molecular and emerging diagnostic approaches for carbapenemase detection and colonisation screening; and (vi) current and emerging therapeutic strategies for carbapenem‐resistant infections.

The included studies were critically compared to identify areas of consensus, conflicting findings, emerging trends and existing knowledge gaps. Particular attention was given to differences in the geographical distribution of carbapenemases, species‐specific resistance mechanisms, diagnostic performance and therapeutic outcomes. Variations arising from differences in study design, patient populations, surveillance intensity, laboratory methodologies and diagnostic criteria were carefully considered during evidence synthesis. Where discrepancies between studies were identified, potential explanations, including regional epidemiological differences, methodological heterogeneity and variations in healthcare infrastructure, were explored. Furthermore, commonly reported limitations of the included studies, such as small sample sizes, regional bias, inconsistent diagnostic approaches and differences in antimicrobial susceptibility testing methods, were evaluated to provide a balanced interpretation of the available evidence.

### 2.8. Quality Assessment and Source Reliability

To ensure the scientific validity and integrity of the synthesised evidence, priority was given to high‐impact, peer‐reviewed literature and official surveillance reports or guidelines issued by recognised international public health authorities. This included publications from the World Health Organization (WHO), the Centers for Disease Control and Prevention (CDC) and the European Centre for Disease Prevention and Control (ECDC). Seminal historical papers and recent high‐quality studies were preferentially selected to balance foundational knowledge with contemporary epidemiological trends.

Consistent with PRISMA‐ScR recommendations, a formal risk‐of‐bias assessment was not undertaken because the primary objective of this scoping review was to map the breadth, nature and extent of the available evidence rather than quantitatively evaluate intervention effectiveness or generate pooled effect estimates. Nevertheless, emphasis was placed on peer‐reviewed studies, recognised surveillance reports and authoritative international guidelines to enhance the reliability and scientific validity of the included evidence.

### 2.9. Limitations

Although extensive efforts were made to ensure a comprehensive and systematic literature search, several limitations should be acknowledged. First, the review did not utilise a prospectively registered review protocol, which may increase the possibility of selection bias. Second, only English‐language publications were included, potentially excluding relevant studies published in other languages. Third, heterogeneity in study designs, diagnostic methodologies and reporting standards limited direct comparability between studies. Fourth, some potentially relevant grey literature and unpublished surveillance data may not have been captured during the search process. Furthermore, because scoping reviews are designed to provide a broad overview of available evidence, the review did not formally assess study quality or perform quantitative meta‐analysis, which may limit conclusions regarding the relative strength of evidence supporting specific diagnostic or therapeutic approaches.

## 3. Carbapenems

### 3.1. Mechanism of Action of Carbapenems

Carbapenems are broad‐spectrum β‐lactam antibiotics and remain among the most potent agents for treating infections caused by MDR Gram‐negative and Gram‐positive bacteria. Their bactericidal activity is mediated through inhibition of bacterial cell wall synthesis. Specifically, carbapenems exhibit high‐affinity binding to essential PBPs, particularly PBP 1 and PBP 2. This interaction inhibits the transpeptidation reactions required for peptidoglycan cross‐linking, resulting in disruption of cell wall integrity, osmotic instability and ultimately bacterial cell lysis (Figure 2) [1].

**FIGURE 2:**
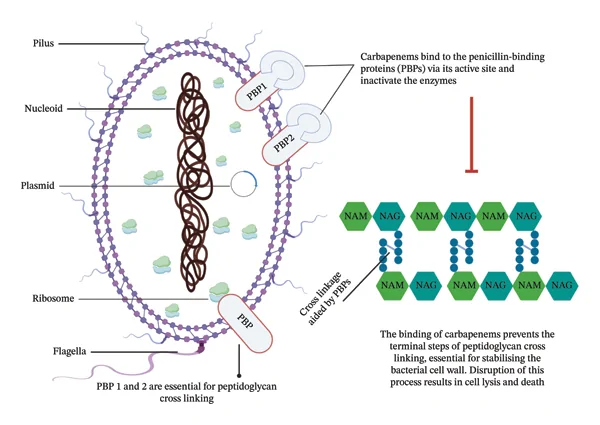
Mechanism of action of carbapenems. Schematic representation illustrating how carbapenems bind with high affinity to essential penicillin‐binding proteins (PBPs), thereby inhibiting the transpeptidation and cross‐linking of N‐acetylglucosamine (NAG) and N‐acetylmuramic acid (NAM) during peptidoglycan synthesis. Disruption of these terminal steps compromises cell wall integrity and leads to bacterial lysis and clearance. Image created with BioRender.

A distinguishing characteristic of carbapenems is their exceptional stability against hydrolysis by many β‐lactamases, including the classical TEM and SHV enzymes that readily inactivate penicillins and cephalosporins. Consequently, carbapenems have historically been regarded as the drugs of choice for severe infections caused by ESBL‐producing Enterobacterales and other MDR Gram‐negative pathogens [2].

The broad antimicrobial spectrum of carbapenems is largely attributable to their unique molecular structure. Unlike other β‐lactam antibiotics, carbapenems possess a trans‐configured hydroxyethyl side chain and a carbon atom replacing the sulphur atom within the five‐membered ring. These structural modifications enhance affinity for PBPs, improve penetration through the outer membrane of Gram‐negative bacteria and confer substantial resistance to degradation by many β‐lactamases [2]. As a result, carbapenems demonstrate potent activity against a wide range of aerobic and anaerobic bacteria, including many organisms resistant to other β‐lactam classes.

The widespread clinical reliance on carbapenems has, however, exerted substantial selective pressure on bacterial populations, contributing to the emergence and global dissemination of CPEs. The evolution of carbapenem resistance has significantly compromised the utility of these agents and represents one of the most pressing challenges in contemporary antimicrobial therapy.

### 3.2. Mechanisms of Resistance to Carbapenems

Despite their broad activity and structural resilience against most β‐lactamases, carbapenem resistance has emerged rapidly, particularly among *Enterobacteriaceae*, *Pseudomonas aeruginosa* and *Acinetobacter baumannii*, and now represents one of the most significant threats to modern antimicrobial therapy. Carbapenem resistance results from a combination of enzymatic and nonenzymatic mechanisms, with the relative contribution of each varying substantially among bacterial species. Although multiple pathways have been described, carbapenemase production remains the predominant mechanism responsible for the global dissemination of CREs.

#### 3.2.1. Carbapenemase Production

Carbapenemases are β‐lactamases capable of hydrolysing carbapenems and most other β‐lactam antibiotics (Figure 3B). These enzymes constitute the most clinically important mechanism of carbapenem resistance because they are frequently encoded on mobile genetic elements, facilitating rapid horizontal transfer within and between bacterial species.

**FIGURE 3:**
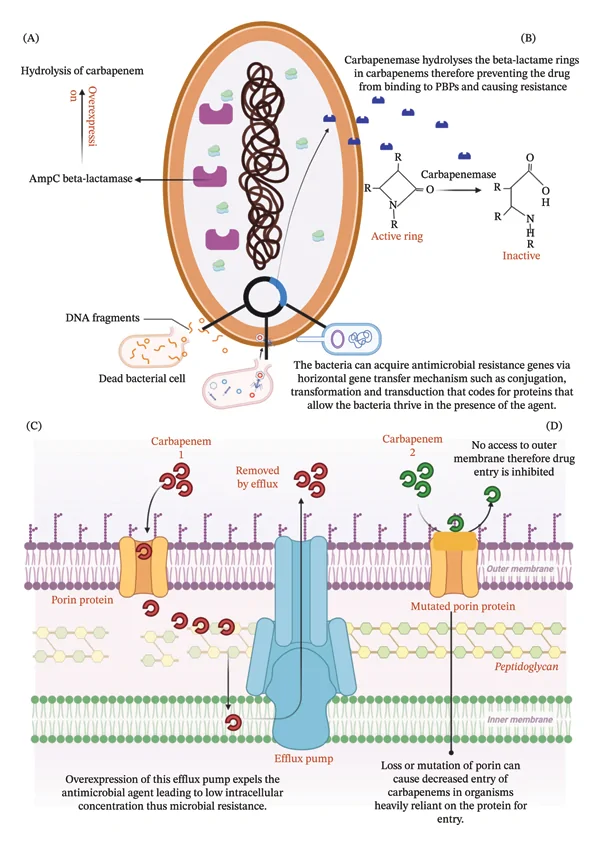
Mechanisms of carbapenem resistance in Gram‐negative bacteria. Schematic representation of the major pathways contributing to carbapenem resistance. (A) Hyperproduction or structural modification of chromosomal AmpC β‐lactamases can contribute to elevated carbapenem minimum inhibitory concentrations, particularly when combined with porin loss. (B) Acquisition of carbapenemase enzymes via horizontal gene transfer (e.g., KPC, NDM, OXA types) results in direct hydrolysis and inactivation of carbapenems. (C) Overexpression of multidrug efflux pumps (e.g., MexAB‐OprM, AdeABC) decreases intracellular antibiotic accumulation, reducing carbapenem efficacy. (D) Loss, downregulation or mutation of outer membrane porins (e.g., OmpK35/36, OprD) restricts carbapenem entry, especially in organisms that simultaneously produce ESBLs or AmpC enzymes. Image created with BioRender.

According to the Ambler classification system, carbapenemases are grouped into Classes A, B and D.

• Class A (Serine Carbapenemases): Mainly represented by KPC enzymes. KPC‐producing organisms are now globally disseminated and frequently associated with highly mobile plasmids, facilitating rapid horizontal and interspecies transfer [12].
• Class B MBLs: These include NDM, VIM and IMP. These enzymes require zinc ions for catalytic activity and are not inhibited by conventional β‐lactamase inhibitors. NDM‐producing *K. pneumoniae* and *E. coli* continue to expand globally and are widely reported in hospital outbreaks and community settings [13].
• Class D carbapenemases (OXA‐type enzymes) (OXAs): These are represented predominantly by OXA‐48‐like enzymes in Enterobacterales and OXA‐23, OXA‐24/40 and OXA‐58 enzymes in *A. baumannii*. Although often chromosomally encoded, their expression is frequently enhanced by insertion sequences such as ISAba1, enabling mobilisation and increased transcription [14].

Although the distribution of KPC enzymes is quite extensive, there is marked variability in the epidemiology of carbapenemases depending on geographical location. In North America and some parts of Europe, for example, KPC enzymes are predominant, while NDM enzymes are more common in South Asia and Africa [15, 16]. This could be due to differences in the use of antibiotics, medical care facilities, global travel and clonal spread [7, 15].

#### 3.2.2. Efflux Pump Overexpression

Multidrug efflux systems actively export antimicrobial agents from the bacterial cell, reducing intracellular drug accumulation. In *P. aeruginosa*, overexpression of the MexAB‐OprM, MexXY and related efflux systems contributes to reduced carbapenem susceptibility. Similarly, the AdeABC, AdeIJK and AdeFGH systems play important roles in multidrug resistance among *A. baumannii* [17].

Although efflux‐mediated resistance generally produces only moderate increases in carbapenem minimum inhibitory concentrations (MICs), it frequently acts synergistically with porin alterations and β‐lactamase production to generate clinically relevant resistance phenotypes (Figure 3C) [4]. Consequently, its contribution is often difficult to quantify independently in clinical isolates.

#### 3.2.3. Porin Loss or Modification

Outer membrane porins facilitate the entry of carbapenems into Gram‐negative bacteria. Reduced permeability resulting from porin loss, structural modification or downregulation can significantly decrease intracellular antibiotic concentrations.

Among Enterobacterales, alterations in OmpK35 and OmpK36 are frequently observed in carbapenem‐resistant *K. pneumoniae*. Although porin loss alone often results in only modest increases in carbapenem MICs, its combination with ESBL or AmpC β‐lactamase production frequently produces clinically significant resistance. Consequently, porin deficiency is considered an important accessory mechanism that enhances the impact of enzymatic resistance pathways (Figure 3D) [18].

In *P. aeruginosa*, imipenem resistance is strongly associated with OprD porin loss, either through mutation, downregulation or deletion [19]. Despite the fact that overexpression of the efflux pump and deficiency in porin proteins are both known factors associated with carbapenem resistance, their role as independent factors is hard to establish quantitatively since they are usually found alongside carbapenemase activity [20, 21]. This means that the majority of studies conducted on the subject have been experimental in nature, investigating either gene expression or MIC alteration in laboratories [20].

#### 3.2.4. AmpC Hyperproduction and Mutational Enzyme Variants

Hyperexpression of chromosomal AmpC β‐lactamases and mutations that expand their substrate spectrum can contribute to carbapenem resistance in several Enterobacterales species, particularly *Enterobacter cloacae* complex, *Citrobacter freundii* and *Serratia marcescens*. However, AmpC hyperproduction alone rarely confers high‐level carbapenem resistance. Rather, clinically significant resistance usually emerges when AmpC overexpression occurs concurrently with porin loss or other permeability defects [5] (Figure 3A).

Although all of the above resistance mechanisms are extensively documented in experimental studies, the significance of each one in contributing to carbapenem resistance in a clinical setting differs between species of bacteria and the healthcare facilities where they occur. In addition, many of the above mechanisms have been studied under laboratory conditions and do not necessarily accurately represent the reality of resistance development in a clinical setting. The predominant resistance mechanisms reported among clinically important CRE are summarised in Table 1.

**TABLE 1:** Predominant carbapenem resistance mechanisms reported among clinically important Enterobacterales.

Species	Predominant carbapenem resistance mechanism	Important secondary mechanisms	Major geographic distribution/comments
*Klebsiella pneumoniae*	KPC, NDM and OXA‐48‐like carbapenemases [7, 22]	OmpK35/OmpK36 porin loss, ESBL production	Major global reservoir of carbapenemase genes; dominant cause of CRE outbreaks worldwide
*Escherichia coli*	NDM and OXA‐48‐like carbapenemases [23, 24]	Porin alterations, ESBL and AmpC production	Increasingly reported in both hospital and community settings
*Enterobacter cloacae* complex	AmpC hyperproduction combined with porin loss [25–28]	KPC and NDM carbapenemases	Frequently associated with healthcare‐associated infections
*Serratia marcescens*	SME and KPC carbapenemases [26]	Reduced outer membrane permeability, AmpC production	Less common but associated with sporadic hospital outbreaks

## 4. Carbapenemases

### 4.1. Historical Perspective

Carbapenemases are β‐lactamases capable of hydrolysing carbapenems and other β‐lactam antibiotics and represent the most clinically significant mechanism of carbapenem resistance among Gram‐negative bacteria. The extensive use of carbapenems for infections caused by ESBL‐ and AmpC‐producing organisms has imposed strong selective pressure, contributing to the emergence and global dissemination of CPOs [29–31].

Carbapenemase genes are frequently located on plasmids, integrons, transposons and other mobile genetic elements, enabling horizontal transfer across species and healthcare settings. This mobility has been central to the global expansion of CPE, particularly *Klebsiella pneumoniae* and *Escherichia coli*. However, the true global burden of CPE remains difficult to estimate because molecular surveillance is unevenly distributed, with substantial underreporting in many low‐ and middle‐income countries [9, 32].

Historically, carbapenemase activity was first recognised in environmental and Gram‐positive organisms, including *Bacillus* spp., before clinically important enzymes were identified in Gram‐negative bacteria. Early carbapenemases in Enterobacterales included NmcA in *Enterobacter cloacae* and SME enzymes in *Serratia marcescens*, many of which were chromosomally encoded and therefore had limited dissemination potential [9, 33].

The epidemiological landscape changed markedly with the emergence of plasmid‐mediated carbapenemases. IMP‐ and VIM‐type MBLs were initially associated with localised outbreaks in Japan and Europe. Subsequently, large‐scale global dissemination occurred following the identification of KPC in the United States, OXA‐48 in Turkey and NDM‐1 in a patient with healthcare exposure in India [33, 34]. Today, KPC, NDM, OXA‐48‐like, VIM and IMP enzymes constitute the principal carbapenemase families driving the global spread of CPE.

Although OXA‐type carbapenemases such as OXA‐23, OXA‐24/40 and OXA‐58 are particularly important in *Acinetobacter baumannii*, OXA‐48‐like enzymes are the most clinically relevant Class D carbapenemases among Enterobacterales. Their relatively weak carbapenem‐hydrolysing activity may complicate phenotypic detection, especially in isolates with low carbapenem MICs, thereby contributing to silent dissemination [6, 35–37].

Overall, the global spread of carbapenemases reflects the combined effects of mobile resistance elements, successful epidemic clones, antimicrobial selection pressure, international travel, healthcare‐associated transmission and inadequate surveillance. Continuous molecular surveillance, rapid detection and timely reporting of emerging carbapenemases remain essential for containment [38].

### 4.2. Classification of Carbapenemases

Carbapenemases may be classified using either the functional Bush–Jacoby–Medeiros system or the molecular Ambler classification. The Bush–Jacoby–Medeiros system categorises β‐lactamases according to substrate profile and inhibitor susceptibility, whereas the Ambler system classifies enzymes according to amino acid sequence homology and active‐site chemistry [39, 40]. A summary of the major classification frameworks and clinically relevant carbapenemase groups is presented in Table 2.

**TABLE 2:** Major classification systems of β‐lactamases and clinically relevant carbapenemase groups.

Classification system	Class/group	Subclass	Enzyme type/description
Ambler (Molecular) Classification	A	—	Serine β‐lactamases, including clinically important carbapenemases such as KPC, SME, IMI and selected GES variants
	B	—	Zinc‐dependent metallo‐β‐lactamases (MBLs), including NDM, VIM and IMP enzymes
	C	—	AmpC cephalosporinases, typically chromosomal or plasmid‐mediated; only rarely associated with carbapenemase activity
	D	—	Oxacillinases (OXA‐type β‐lactamases), including OXA‐48‐like and other carbapenem‐hydrolysing OXA enzymes

Bush–Jacoby–Medeiros (Functional) Classification	Group 1	—	Cephalosporinases (AmpC‐type enzymes) that are generally resistant to clavulanic acid inhibition
	Group 2a	2a	Penicillinases predominantly found in Gram‐positive bacteria
	Group 2b	2b	Broad‐spectrum β‐lactamases inhibited by clavulanic acid or tazobactam
	Group 2c	2c	Carboxypenicillinases with enhanced hydrolysis of carbenicillin and ticarcillin
	Group 2d	OXA	OXA‐type β‐lactamases, some of which possess carbapenemase activity
	Group 2e	2e	Extended‐spectrum cephalosporinases with activity against oxyimino‐cephalosporins
	Group 2f	2f	Serine carbapenemases, including KPC, IMI, NmcA and SME enzymes
	Group 3	—	Zinc‐dependent metallo‐β‐lactamases (MBLs), including NDM, VIM and IMP enzymes
	Group 4	—	Rare and poorly characterised β‐lactamases; largely omitted from the 2010 update because of limited biochemical data

#### 4.2.1. Functional (Bush–Jacoby–Medeiros) Classification

In the Bush–Jacoby–Medeiros system, β‐lactamases are divided into functional groups based on hydrolytic activity and inhibitor profile. Group 1 enzymes are mainly cephalosporinases, including AmpC β‐lactamases, which are generally resistant to inhibition by clavulanic acid. Group 2 enzymes comprise serine β‐lactamases and include several clinically important subgroups. Subgroup 2f contains serine carbapenemases such as KPC, SME, IMI and NmcA, whereas Subgroup 2d includes OXA‐type enzymes, some of which possess carbapenem‐hydrolysing activity. Group 3 enzymes are MBLs, including NDM, VIM and IMP, which require zinc ions for activity and hydrolyse most β‐lactams except monobactams such as aztreonam. Group 4 enzymes are rare and poorly characterised and were largely omitted from later revisions because of limited biochemical data [9, 39–41].

#### 4.2.2. Molecular (Ambler) Classification

The Ambler classification divides β‐lactamases into four molecular classes. Classes A, C and D are serine β‐lactamases, whereas Class B enzymes are zinc‐dependent MBLs. Clinically important carbapenemases occur mainly in Ambler Classes A, B and D, while Class C enzymes usually contribute to carbapenem resistance indirectly through AmpC hyperproduction combined with permeability defects.

##### 4.2.2.1. Class A Carbapenemases

Class A carbapenemases are serine β‐lactamases capable of hydrolysing penicillins, cephalosporins, aztreonam and carbapenems. They are variably inhibited by clavulanate, tazobactam, avibactam, vaborbactam, relebactam and boronic acid derivatives, depending on the specific enzyme [42].

Chromosomally encoded Class A carbapenemases, including NmcA, SME, IMI and SFC‐1, are relatively uncommon and have limited dissemination. In contrast, plasmid‐mediated KPC enzymes are the most clinically important Class A carbapenemases because of their extensive global spread, especially among *K. pneumoniae* epidemic lineages such as ST258, ST11 and related high‐risk clones [9, 43, 44]. KPC enzymes are now reported worldwide and are often associated with multidrug resistance, porin alterations and mobile genetic elements that facilitate interspecies transfer [45, 46].

GES‐type enzymes were initially described as ESBLs, but some variants, including GES‐2, GES‐5, GES‐11 and GES‐20, possess carbapenemase activity. However, GES carbapenemases remain less prevalent than KPC and are generally associated with sporadic outbreaks or regional dissemination [47, 48].

##### 4.2.2.2. Class B Carbapenemases: MBLs

Class B carbapenemases are zinc‐dependent MBLs that hydrolyse nearly all β‐lactams, including carbapenems, but generally spare aztreonam. They are not inhibited by classical β‐lactamase inhibitors such as clavulanate, tazobactam, avibactam, relebactam or vaborbactam. Their dependence on zinc explains their inhibition by metal chelators such as EDTA, although such inhibitors are not clinically useful.

The most important acquired MBL families include NDM, VIM and IMP. IMP‐type enzymes were first described in Japan and are now reported globally. VIM enzymes were initially recognised in Europe and remain important in both *Pseudomonas aeruginosa* and Enterobacterales. NDM enzymes have become particularly important in Enterobacterales, especially *K. pneumoniae* and *E. coli*, and are now widely distributed across South Asia, the Middle East, Africa, Europe and other regions [30, 49–51].

MBL‐producing Enterobacterales are especially difficult to treat because most currently approved β‐lactam/β‐lactamase inhibitor combinations lack activity against MBLs. This distinguishes them from KPC‐ and OXA‐48‐producing isolates, for which newer inhibitor combinations have substantially improved treatment options. The global spread of MBLs, their frequent co‐carriage with resistance determinants to aminoglycosides, fluoroquinolones and colistin, and the absence of widely available MBL inhibitors make them a major therapeutic and surveillance priority.

##### 4.2.2.3. Class D Carbapenemases: OXA‐type Enzymes

Class D β‐lactamases, commonly referred to as OXAs, are serine enzymes originally characterised by their ability to hydrolyse oxacillin and related penicillins. Among Enterobacterales, OXA‐48‐like enzymes are the most clinically important Class D carbapenemases. First identified in Turkey, OXA‐48‐like enzymes are now widely reported across Europe, North Africa, the Middle East and increasingly other regions [46–48].

OXA‐48‐like enzymes often confer low‐level carbapenem resistance and may not substantially increase carbapenem MICs in the absence of additional resistance mechanisms. However, when combined with ESBLs, AmpC enzymes or porin defects, they can produce clinically significant resistance. Their weak carbapenem‐hydrolysing phenotype also creates diagnostic challenges, particularly in laboratories relying solely on routine susceptibility testing [52–54].

In *A. baumannii*, OXA‐23‐like, OXA‐24/40‐like, OXA‐58‐like, OXA‐143‐like and OXA‐235‐like enzymes are major contributors to carbapenem resistance. These enzymes are clinically important in nonfermenting Gram‐negative pathogens but should be interpreted separately from OXA‐48‐like enzymes, which are the major OXA carbapenemases in Enterobacterales.

##### 4.2.2.4. Class C β‐Lactamases and Carbapenem Resistance

Class C β‐lactamases, also known as AmpC cephalosporinases, are not typically classified as major carbapenemases. However, AmpC hyperproduction can contribute to carbapenem resistance when combined with porin loss or reduced outer membrane permeability, particularly in *Enterobacter cloacae* complex, *Citrobacter freundii* and other AmpC‐producing Enterobacterales [55].

Some AmpC variants, including CMY, ACT and DHA derivatives, have shown enhanced carbapenem‐hydrolysing activity. Nevertheless, their intrinsic carbapenemase activity is generally weak, and their clinical importance usually depends on concurrent permeability defects. Therefore, Class C enzymes are best regarded as contributors to carbapenem resistance rather than principal carbapenemases.

Overall, the Ambler classification is particularly useful for molecular epidemiology and therapeutic decision‐making, whereas the Bush–Jacoby–Medeiros system provides functional insight into substrate profiles and inhibitor susceptibility. Both frameworks remain relevant, but neither fully captures the clinical complexity created by coexisting resistance mechanisms, mobile genetic elements and regional variation in carbapenemase distribution.

### 4.3. Carbapenemase Spread Among Gram‐Negative Pathogens

#### 4.3.1. CPE

Enterobacterales are common colonisers of the human and animal gastrointestinal tract and include major pathogens such as *K. pneumoniae*, *E. coli*, *Enterobacter cloacae* complex, *Citrobacter freundii* and *Serratia marcescens*. These organisms are frequent causes of urinary tract infections (UTIs), pneumonia, intra‐abdominal infections, bloodstream infections (BSIs) and healthcare‐associated outbreaks. Their ability to acquire and exchange plasmids has made them central drivers of global antimicrobial resistance dissemination [9, 56].

CPE pose a major public health threat because carbapenemase genes are often carried on mobile elements that also harbour resistance determinants to fluoroquinolones, aminoglycosides, sulphonamides and other antimicrobial classes [57]. As a result, CPE infections are frequently associated with delayed effective therapy, limited treatment options, prolonged hospitalisation and increased mortality.

The distribution of carbapenemase families varies geographically. KPC enzymes remain highly prevalent in North America, parts of South America, Southern Europe, Israel and China. NDM enzymes are especially common in South Asia and are increasingly reported across Africa, the Middle East and Europe. OXA‐48‐like enzymes are strongly associated with Turkey, North Africa, the Middle East and parts of Europe. VIM and IMP enzymes are more regionally variable but remain important contributors to carbapenem resistance in both Enterobacterales and nonfermenting Gram‐negative organisms [28, 58, 59].

Although carbapenemase production is the dominant mechanism in CPE, additional mechanisms often amplify resistance. Porin loss, such as OmpK35/OmpK36 alterations in *K. pneumoniae* and OmpF/OmpC changes in *E. coli*, reduces carbapenem entry. Efflux systems such as AcrAB‐TolC may further reduce intracellular drug accumulation. These mechanisms are especially important when combined with ESBL or AmpC production [60–67].

The true global prevalence of CPE remains uncertain. Reported estimates vary because of differences in surveillance intensity, diagnostic capacity, sampling populations and reporting systems. Many studies are derived from tertiary hospitals with high‐risk patients, which may overestimate prevalence relative to community settings. Conversely, limited molecular surveillance in many low‐ and middle‐income countries likely leads to substantial underestimation of CPE burden [68, 69]. The global epidemiology of these isolates and resistant enzymes is presented in Table 3.

**TABLE 3:** Regional distribution and epidemiological trends of major carbapenemases among carbapenemase‐producing Enterobacterales.

Region	Predominant carbapenemase(s)	Major species reported	Epidemiological trend	References
North America	KPC	*K. pneumoniae*	Endemic in many healthcare settings	[7]
South Asia	NDM	*E. coli*, *K. pneumoniae*	Rapidly increasing and widely disseminated	[22, 70]
Europe	KPC, OXA‐48‐like	*K. pneumoniae*	Heterogeneous, with marked country‐level variation	[71, 72]
Middle East and North Africa	OXA‐48‐like, NDM	Enterobacterales	High prevalence in several countries	[23, 73]
China	KPC, NDM	*K. pneumoniae*, *E. coli*	Expanding, with increasing regional diversity	[74, 75]
Sub‐Saharan Africa	NDM, OXA‐48‐like	Enterobacterales	Likely underreported due to limited molecular surveillance	[76, 77]

#### 4.3.2. Other Carbapenem‐Resistant Gram‐Negative Organisms

Although this review focuses primarily on CRE and CPE, carbapenem resistance in nonfermenting Gram‐negative pathogens such as *P. aeruginosa* and *A. baumannii* remains clinically important. These organisms are frequent causes of healthcare‐associated pneumonia, BSIs, wound infections and intensive care unit outbreaks.

In *P. aeruginosa*, carbapenem resistance commonly results from OprD porin loss, efflux pump overexpression, AmpC overproduction and, in some regions, acquisition of VIM, IMP or other carbapenemases. In *A. baumannii*, carbapenem resistance is most commonly associated with OXA‐type carbapenemases, particularly OXA‐23‐like enzymes, often combined with efflux and permeability changes [6, 7]. These organisms may act as reservoirs of resistance determinants and contribute to the broader ecology of carbapenem resistance, but their epidemiology and resistance biology differ substantially from Enterobacterales (see Table 4).

**TABLE 4:** Comparative overview of major carbapenem‐resistant Gram‐negative pathogens, resistance mechanisms and clinically relevant carbapenemases.

Pathogen	Common clinical infections	Major resistance mechanisms	Predominant carbapenemases	Clinical and epidemiological significance
*Enterobacterales* (*K. pneumoniae*, *E. coli*, *Enterobacter* spp.)	Urinary tract infections, bloodstream infections, pneumonia, intra‐abdominal infections	Carbapenemase production, ESBL or AmpC co‐production, porin loss (OmpK35/OmpK36, OmpF/OmpC), efflux pump overexpression	KPC, NDM, OXA‐48‐like, VIM, IMP	Principal drivers of the global CRE epidemic. *K. pneumoniae* ST258, ST11 and related high‐risk clones facilitate widespread plasmid‐mediated dissemination of carbapenemase genes.
*Pseudomonas aeruginosa*	Ventilator‐associated pneumonia, sepsis, burn wound infections, chronic respiratory infections	OprD porin loss, efflux pump overexpression (MexAB‐OprM and related systems), AmpC hyperproduction, carbapenemase acquisition	VIM, IMP, NDM, GES, KPC (less common)	Important healthcare‐associated pathogen and reservoir of metallo‐β‐lactamases. Frequently associated with multi–drug‐resistant and extensively drug‐resistant phenotypes.
Acinetobacter baumannii	Ventilator‐associated pneumonia, bloodstream infections, wound infections, meningitis	OXA‐type carbapenemases, efflux pump activation, porin alterations, AmpC overexpression	OXA‐23‐like, OXA‐24/40‐like, OXA‐58‐like, NDM	Major cause of intensive care unit outbreaks. OXA‐23‐like enzymes are globally dominant and contribute substantially to carbapenem resistance.
Other Gram‐negative organisms (Burkholderia cepacia complex, *Raoultella* spp., environmental species)	Opportunistic respiratory and device‐associated infections	Environmental reservoirs, plasmid‐mediated resistance, horizontal gene transfer	PenA, KPC, NDM, other emerging carbapenemases	Emerging reservoirs of carbapenemase genes within environmental and healthcare ecosystems, contributing to resistance dissemination.

#### 4.3.3. Major Infections and Diseases Caused by CPE

CPEs, particularly *Escherichia coli* and *Klebsiella pneumoniae*, are associated with a wide spectrum of community‐onset and healthcare‐associated infections. Their clinical impact reflects not only carbapenem resistance but also frequent coresistance to other antimicrobial classes, which limits empirical and definitive treatment options.

##### 4.3.3.1. UTIs

CPE are increasingly implicated in complicated UTIs, including catheter‐associated infection, pyelonephritis and urosepsis. Patients with diabetes, recurrent UTI, urinary catheterisation, structural urological abnormalities or recent healthcare exposure are at increased risk. Multidrug resistance frequently limits oral treatment options and increases the risk of recurrence or progression to invasive disease [78].

##### 4.3.3.2. BSIs

CPE are important causes of BSI and sepsis, particularly among critically ill, immunocompromised and hospitalised patients with invasive devices. Carbapenemase‐producing *K. pneumoniae*, including KPC‐, NDM‐ and OXA‐48‐producing strains, is especially associated with severe BSI. Mortality is often high, particularly when active therapy is delayed or source control is inadequate [79].

##### 4.3.3.3. Pneumonia and Lower Respiratory Tract Infections

CPE contribute to hospital‐acquired pneumonia and ventilator‐associated pneumonia, especially in intensive care settings. Risk factors include mechanical ventilation, prolonged hospitalisation, prior broad‐spectrum antibiotic exposure and severe underlying illness. These infections are difficult to manage because of high bacterial burden, limited antibiotic penetration at the infection site and frequent coresistance [79].

##### 4.3.3.4. Intra‐Abdominal Infections

CPE can cause severe intra‐abdominal infections, including peritonitis, cholecystitis, postoperative intra‐abdominal sepsis and abscesses. These infections often occur after gastrointestinal surgery, bowel perforation or in immunocompromised patients. Effective management usually requires both antimicrobial therapy and adequate source control [12].

##### 4.3.3.5. Central Nervous System Infections

Although uncommon, CPE‐associated meningitis and ventriculitis have been reported, particularly after neurosurgery, head trauma or ventricular shunt placement. Management is challenging because many potentially active agents have limited central nervous system penetration, and clinical outcomes are often poor [80].

## 5. Molecular Detection Methods for CP‐CRE

The accurate detection of CPE is essential for guiding antimicrobial therapy, implementing infection prevention measures and supporting surveillance programmes. Detection remains challenging because of the genetic diversity of carbapenemases, the variable phenotypic expression of resistance and the continual emergence of novel enzyme variants. While phenotypic methods remain valuable for initial screening, molecular diagnostics provide definitive identification of carbapenemase genes and are increasingly incorporated into routine clinical microbiology workflows.

Phenotypic assays such as the modified carbapenem inactivation method (mCIM), EDTA‐modified eCIM, Carba NP test and broth microdilution remain widely used because of their relative simplicity and cost‐effectiveness. Broth microdilution remains the reference method for antimicrobial susceptibility testing and determination of carbapenem MICs [81]. However, phenotypic assays cannot reliably identify specific carbapenemase genes and may demonstrate variable performance depending on the enzyme present. For example, OXA‐48‐like carbapenemases often exhibit relatively weak carbapenem‐hydrolysing activity and may therefore generate false‐negative or ambiguous results in some phenotypic assays [82, 83].

Polymerase chain reaction (PCR)–based methods remain the most widely adopted molecular tools for detecting common carbapenemase genes, including *bla*KPC, *bla*NDM, *bla*VIM, *bla*IMP and *bla*OXA‐48‐like. These assays offer excellent sensitivity and specificity and can provide results within a few hours [44]. However, PCR cannot detect uncharacterised genes and requires laboratory infrastructure. However, PCR assays are inherently target‐dependent and may fail to detect novel or previously uncharacterised carbapenemase genes. Consequently, exclusive reliance on PCR‐based diagnostics may contribute to underrecognition of emerging resistance mechanisms.

Several rapid molecular platforms have been developed to improve accessibility and turnaround time. Cartridge‐based systems such as Xpert Carba‐R permit direct detection of major carbapenemase genes from clinical specimens within approximately 1 hour and have demonstrated high diagnostic accuracy. Loop‐mediated isothermal amplification (LAMP) assays represent an alternative molecular approach that combines rapid detection with minimal equipment requirements, making them attractive for resource‐limited settings [82, 83].

Rapid immunochromatographic lateral‐flow assays have emerged as valuable adjuncts for carbapenemase detection. Commercial platforms such as NG‐Test CARBA 5 enable simultaneous identification of the five major carbapenemase families (KPC, NDM, VIM, IMP and OXA‐48‐like enzymes) directly from bacterial colonies within a short duration. Their ease of use, rapid turnaround time and high sensitivity make them particularly useful for infection control screening and outbreak investigations.

Microarray‐based technologies allow simultaneous detection of multiple resistance determinants and β‐lactamase families. Although highly informative, their adoption remains limited by cost and specialised laboratory requirements. Whole‐genome sequencing (WGS) currently represents the most comprehensive diagnostic approach. In addition to identifying known and novel carbapenemase genes, WGS can characterise plasmid structures, track transmission pathways, identify coresistance determinants and support molecular epidemiological investigations during outbreaks [84]. Nevertheless, widespread implementation remains constrained by financial costs, infrastructure requirements, data analysis complexity and the need for specialised bioinformatics expertise.

Active surveillance for asymptomatic CPE colonisation is a critical component of infection prevention and control programmes. Gastrointestinal colonisation frequently precedes infection and serves as an important reservoir for healthcare‐associated transmission. Rectal swabs remain the preferred specimen for colonisation screening and may be analysed using selective chromogenic media, molecular assays or rapid immunochromatographic tests.

Targeted screening is commonly performed in high‐risk patient populations, including intensive care unit admissions, patients transferred from healthcare facilities with known CPE outbreaks and individuals with recent international healthcare exposure. Early identification of colonised patients facilitates timely implementation of contact precautions and reduces nosocomial transmission.

### 5.1. Emerging and Adjunct Diagnostic Approaches

Several innovative diagnostic strategies are currently under investigation.

a. Macrophage‐based detection platforms utilise host immune cell responses combined with nanotechnology‐based detection systems to identify bacterial virulence signatures and antimicrobial susceptibility patterns. Although still experimental, these approaches may enable rapid functional assessment of bacterial pathogens [85].
b. Phage‐based diagnostics exploit the high specificity of bacteriophages and phage‐derived proteins for bacterial targets. Engineered reporter phages and endolysin‐based detection systems have demonstrated promising diagnostic potential for rapid identification of viable bacterial populations and antimicrobial resistance determinants.
c. Reactive oxygen species (ROS)–based biosensors utilise oxidative stress signatures generated during bacterial growth and biofilm formation. These systems may provide simultaneous diagnostic and antimicrobial functions, particularly in biofilm‐associated infections [86].

Despite substantial technological advances, important challenges remain. Most diagnostic performance studies have been conducted in well‐resourced healthcare settings, limiting generalisability to low‐resource environments. Furthermore, differences in specimen types, study populations, carbapenemase prevalence and reference standards complicate direct comparisons across studies. Current evidence therefore supports a complementary diagnostic strategy that combines phenotypic screening with targeted molecular confirmation rather than reliance on any single diagnostic platform (see Table 5).

**TABLE 5:** Comparison of diagnostic methods for carbapenemase detection.

Method	Turnaround time	Detects enzyme activity	Identifies carbapenemase type	Advantages	Limitations
Broth microdilution	18–24 h	No	No	Gold standard susceptibility testing	No mechanism identification
Carba NP	< 2 h	Yes	No	Rapid and inexpensive	Lower sensitivity for OXA‐48
mCIM/eCIM	18–24 h	Yes	Partial	Differentiates MBLs	Labour‐intensive
Lateral‐flow immunoassays	15–30 min	Yes	Yes	Detects KPC, NDM, VIM, IMP, OXA‐48 directly	Limited to known targets
PCR	2–6 h	No	Yes	High sensitivity and specificity	Cannot detect novel genes
LAMP	< 1 h	No	Yes	Rapid and suitable for low‐resource settings	Target‐dependent
WGS	24–72 h	Yes	Yes	Comprehensive genomic information	Expensive and technically demanding

## 6. Emerging Therapeutic Strategies and Clinical Outcomes

The treatment of infections caused by CPEs remains challenging because carbapenemase genes frequently coexist with resistance determinants targeting aminoglycosides, fluoroquinolones, sulphonamides and polymyxins. Delayed administration of effective therapy is associated with significantly increased mortality, particularly in BSIs and severe hospital‐acquired pneumonia [86].

Historically, polymyxins, tigecycline and aminoglycosides were frequently used as salvage therapies. However, concerns regarding nephrotoxicity, suboptimal pharmacokinetics and inconsistent clinical efficacy have limited their utility [87–91]. Increasing evidence suggests that monotherapy with polymyxins is associated with poorer outcomes than appropriately selected combination regimens [92–95].

The introduction of novel β‐lactam/β‐lactamase inhibitor combinations has transformed the management of KPC‐producing infections. Ceftazidime–avibactam, meropenem–vaborbactam and imipenem–cilastatin–relebactam exhibit potent activity against KPC‐producing Enterobacterales and are currently considered preferred therapeutic options in many treatment guidelines [96–105].

Among these agents, ceftazidime–avibactam has demonstrated favourable clinical outcomes across multiple infection types, including BSIs and complicated UTIs [98–100]. However, resistance emerging during therapy has increasingly been reported, particularly among prolonged or recurrent infections [101–105].

OXA‐48‐like carbapenemases present unique therapeutic challenges because they often coexist with ESBLs and may evade routine detection. Unlike MBLs, OXA‐48 enzymes are inhibited by avibactam. Consequently, ceftazidime–avibactam remains one of the preferred treatment options for OXA‐48‐producing Enterobacterales.

MBL‐producing organisms, including NDM‐, VIM‐ and IMP‐producing Enterobacterales, represent some of the most difficult carbapenemase‐producing pathogens to treat. Classical β‐lactamase inhibitors, including avibactam, relebactam and vaborbactam, do not inhibit MBLs. The combination of aztreonam and ceftazidime–avibactam has emerged as an important therapeutic strategy because aztreonam remains intrinsically stable to MBL hydrolysis, while avibactam inhibits accompanying serine β‐lactamases that would otherwise inactivate aztreonam. Aztreonam–avibactam, currently under advanced clinical development, represents a particularly promising option for MBL‐producing Enterobacterales [104, 105].

Cefiderocol is a novel siderophore cephalosporin that exploits bacterial iron uptake systems to facilitate intracellular drug transport. Unlike most β‐lactam antibiotics, cefiderocol retains activity against many KPC‐, OXA‐48‐ and MBL‐producing Enterobacterales, as well as carbapenem‐resistant *Pseudomonas aeruginosa* and *Acinetobacter baumannii* [87, 88]. Its broad activity against multiple carbapenemase classes has positioned cefiderocol as an important treatment option for highly resistant Gram‐negative infections. Nevertheless, concerns remain regarding resistance emergence during therapy and variability in clinical outcomes across different infection syndromes [89–91].

Overall, interpretation of therapeutic outcomes remains challenging because available evidence is dominated by observational studies, retrospective analyses and heterogeneous patient populations. Factors such as infection severity, comorbidities, source control and timing of effective therapy substantially influence clinical outcomes. While novel therapeutic agents have significantly expanded treatment options, the continued emergence of resistance underscores the need for ongoing surveillance and antimicrobial innovation.

## 7. Conclusion

CRE represent one of the most significant threats to modern antimicrobial therapy. The global dissemination of carbapenemase families including KPC, NDM, VIM, IMP and OXA‐48‐like enzymes has substantially reduced the effectiveness of carbapenems and contributed to increased morbidity, mortality and healthcare costs worldwide. Although carbapenemase production remains the dominant resistance mechanism, additional factors such as porin alterations, efflux pump overexpression and AmpC hyperproduction frequently amplify resistance phenotypes.

The spread of carbapenem resistance extends beyond healthcare settings and is increasingly linked to environmental reservoirs, wastewater systems, agricultural sources and interspecies transfer of mobile genetic elements. Consequently, containment of carbapenem resistance requires coordinated surveillance strategies that integrate clinical, environmental and public health perspectives.

Significant advances in molecular diagnostics, rapid screening technologies and novel antimicrobial agents have improved the detection and management of CPE. However, major disparities persist in diagnostic capacity, surveillance infrastructure and access to modern therapeutics, particularly in low‐ and middle‐income countries where the burden may be substantially underestimated.

Despite encouraging therapeutic advances, the emergence of resistance to newly introduced β‐lactam/β‐lactamase inhibitor combinations highlights the dynamic nature of antimicrobial resistance and the limitations of relying solely on antibiotic development. Future priorities should include expansion of molecular surveillance networks, wider implementation of rapid diagnostic technologies, optimisation of carbapenemase‐targeted treatment strategies and accelerated development of innovative approaches such as phage therapy, antimicrobial peptides and next‐generation β‐lactamase inhibitors.

Addressing the global challenge of carbapenem resistance will require sustained collaboration among clinicians, microbiologists, researchers, public health authorities and policymakers. Strengthening surveillance, improving diagnostic accessibility and ensuring equitable access to effective therapies will be critical to preserving the utility of existing antimicrobials and mitigating the continued global expansion of carbapenem‐resistant pathogens.

## Author Contributions

Oluchi Judith Osuala: conceptualisation and writing–original draft. Chioma Miracle Ojiako: investigation, visualisation and writing–review and editing. Chioma C. Ezeuko: conceptualisation and visualisation. Kasso Terhemen: writing–review and editing. Constance Chinyere Ezemba: writing–review and editing. Victor O. Etikudike: writing–review and editing. Charles O. Nnadi: writing–review and editing. Angus Nnamdi Oli: conceptualisation and methodology.

## Funding

Open access publishing facilitated by University of the Sunshine Coast, as part of the Wiley ‐ University of the Sunshine Coast agreement via the Council of Australasian University Librarians.

## Ethics Statement

The authors have nothing to report.

## Consent

The authors have nothing to report.

## Conflicts of Interest

The authors declare no conflicts of interest.

## Data Availability

Data sharing is not applicable to this article as no datasets were generated or analysed during the current study.
